# Strong Coupling Dynamics of a Quantum Emitter near a Topological Insulator Nanoparticle

**DOI:** 10.3390/nano13202787

**Published:** 2023-10-18

**Authors:** Ioannis Thanopulos, Vassilios Yannopapas, Emmanuel Paspalakis

**Affiliations:** 1 Materials Science Department, School of Natural Sciences, University of Patras, 265 04 Patras, Greece; paspalak@upatras.gr; 2 Department of Physics, National Technical University of Athens, 157 80 Athens, Greece; vyannop@mail.ntua.gr

**Keywords:** spontaneous emission, topological insulator, two-level quantum system, Purcell effect, strong coupling, non-Markovianity measure, quantum speed limit

## Abstract

We study the spontaneous emission dynamics of a quantum emitter near a topological insulator Bi${}_{2}$Se${}_{3}$ spherical nanoparticle. Using the electromagnetic Green’s tensor method, we find exceptional Purcell factors of the quantum emitter up to $10^{10}$ at distances between the emitter and the nanoparticle as large as half the nanoparticle’s radius in the terahertz regime. We study the spontaneous emission evolution of a quantum emitter for various transition frequencies in the terahertz and various vacuum decay rates. For short vacuum decay times, we observe non-Markovian spontaneous emission dynamics, which correspond perfectly to values of well-established measures of non-Markovianity and possibly indicate considerable dynamical quantum speedup. The dynamics turn progressively Markovian as the vacuum decay times increase, while in this regime, the non-Markovianity measures are nullified, and the quantum speedup vanishes. For the shortest vacuum decay times, we find that the population remains trapped in the emitter, which indicates that a hybrid bound state between the quantum emitter and the continuum of electromagnetic modes as affected by the nanoparticle has been formed. This work demonstrates that a Bi${}_{2}$Se${}_{3}$ spherical nanoparticle can be a nanoscale platform for strong light–matter coupling.

## 1. Introduction

In the last decade, the strong coupling of quantum emitters (QEMI) with photonic microstructures and nanostructures pushes the boundaries of cavity quantum electrodynamics in new regimes and may lead to numerous important phenomena in nanophotonics and applications in quantum technologies [1,2,3,4]. A basic phenomenon under the strong coupling of a QEMI with its nanophotonic surroundings is the exchange of energy between the QEMI and the photonic macrostructure coherently, leading to spontaneous emission (SPEM) dynamics from the QEMI, which are non-Markovian and reversible. This phenomenon has been predicted when a QEMI is coupled to various nanophotonic platforms, including plasmonic nanostructures [5,6,7,8,9,10,11,12,13,14,15,16], epsilon-and-mu-near-zero media [17], two-dimensional semiconductors [18,19,20,21,22], graphene nanostructures [23,24], and ferromagnetic or ferrimagnetic nanoparticles [25]. Another class of photonic structures that have the ability to lead to strong light–matter interaction with QEMIs is born through the merger of quantum optics with topological photonics [26,27,28], and is realized by coupling QEMIs with topological photonic structures, like topological one-dimensional waveguides [29], three-dimensional photonic Weyl environments [30], topological photonic crystals [31,32], and a plasmonic nanoantenna, for on-resonance operation, embedded in a topological photonic structure designed for this purpose [33].

Recently, initial evidence that topological insulator (TOPIN) [34,35] nanoparticles can lead to strong light–matter coupling at the nanoscale has been given [36]. Here, we continue this study, and explore TOPIN spherical nanoparticles (SNPs) in order to obtain strong light–matter interaction strength and non-Markovian SPEM between a QEMI and a TOPIN SNP in the terahertz regime. This strong light–matter interaction is a result of the huge polarization currents supported at the surface of TOPIN materials, due to the colossal values of the dielectric function in the THz regime. Specifically, we consider an SNP made of a TOPIN, which is a bulk band gap electronic material, like a common insulator, but it features protected conducting states on its edge or surface too [34,35]. Recently, the interaction of light with TOPIN microstructures and nanostructures has shown interesting optical properties [37,38,39,40]. In addition, studies of quantum optical effects in coupled systems composed of QEMIs and TOPIN nanostructures and microstructures have been presented [41,42,43,44,45].

In this work, we study the SPEM properties of a QEMI near a Bi${}_{2}$Se${}_{3}$ SNP. We first calculate the QEMI Purcell factor near SNPs with radii between 40 nm and 100 nm using experimental parameters for describing the optical properties of the TOPIN material. We find exceptional Purcell factors of the QEMI up to $10^{10}$ at the terahertz regime and at distances between the QEMI and the SNP as large as half the SNP radius, as well as Purcell factors at least $10^{5}$ over the whole frequency range 1–20 THz. We then analyze the SPEM dynamics of a QEMI for various transition frequencies in the terahertz regime of the spectrum and for vacuum decay times in the ns to ms range, using the population evolution and standard measures of non-Markovianity, as well as the quantum speed limit (QSL). For short vacuum decay times, we observe non-Markovian SPEM dynamics, which correspond perfectly to the obtained values of the measures of non-Markovianity and possibly indicate considerable dynamical quantum speedup. The dynamics turn progressively Markovian as the vacuum decay times increase, while the non-Markovianity measures are nullified and the quantum speedup vanishes. For the shortest free-space decay times, we find that the population remains trapped in the QEMI, which indicates that a hybrid bound state between the QEMI and the continuum of electromagnetic modes as affected by the SNP has been formed. This work demonstrates that a Bi${}_{2}$Se${}_{3}$ SNP can be an important nanoscale platform for strong light–matter coupling in the terahertz regime of the spectrum.

This paper is organized as follows: In Section 2, we present the methodology for investigating the SPEM dynamics of a QEMI next to a TOPIN SNP. In the next section, we present our results on the Purcell enhancement factors of such a QEMI obtained by a first-principle electromagnetic method, and the results on the corresponding SPEM dynamics, including values of standard measures of non-Markovianity and the possible quantum speedup of the dynamics. We finally conclude in Section 4.

## 2. Theory

**Figure 1:**
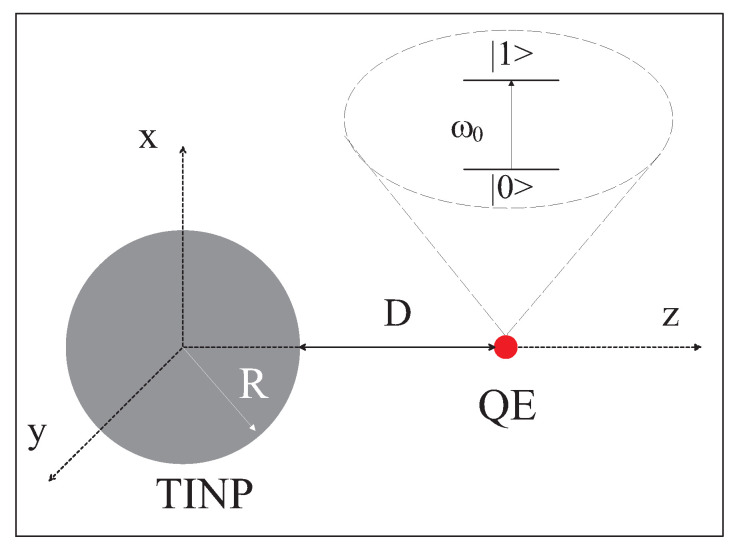
(color online) Schematic depiction of a TOPIN SNP, a Bi${}_{2}$Se${}_{3}$ sphere of radius *R*, in proximity to a two-level QEMI, as in this work.

We study the non-Markovian dynamics of an one-photon SPEM of a QEMI next to a Bi${}_{2}$Se${}_{3}$ SNP of radius *R*. An SNP-centered coordinate system is in use, as shown in Figure 1, where a QEMI placed at ${\tilde{r}}_{\mathrm{QE}}=\left(0,0,R+D\right)$ is shown, too.

The system state at time *t* reads (ℏ=1 is in use in this paper)

(1) $$|\Psi (t)\rangle =c_{1}\left(t\right)e^{-i\omega_{0}t}|1;0_{\omega }\rangle +\int d\vec{r}\int d\omega \;c\left(\vec{r},\omega ,t\right)e^{-i\omega t}|0;1_{\vec{r},\omega }\rangle \;.$$

In Equation (1), |n;a〉=|n〉⊗|a〉 stands for the states of the two-level quantum system (see Figure 1), where |n〉(n=0,1), and |a〉 denotes the states of the photonic environment of the altered photonic modes due to the proximity to the TOPIN SNP, with the vacuum given by $|0_{\omega }\rangle$ and the one-photon state by $|1_{\vec{r},\omega }\rangle$. The equation for $c_{1}\left(t\right)$ reads [9]

(2) $$\begin{array}{ccc} {\dot{c}}_{1}\left(t\right) & = & i\int_{0}^{t}K\left(t-t^{\prime }\right)c_{1}\left(t^{\prime }\right)dt^{\prime }\;, \end{array}$$

(3) $$\begin{array}{ccc} K(t-t^{\prime }) & = & ie^{i\omega_{0}\left(t-t^{\prime }\right)}\int_{0}^{\infty }J\left(\omega \right)e^{-i\omega (t-t^{\prime })}d\omega \;, \end{array}$$

(4) $$\begin{array}{ccc} J(\omega ) & = & \Gamma_{0}\left(\omega_{0}\right)\lambda^{k}\left(\omega ,D\right)/2\pi \; \end{array}$$

with $\Gamma_{0}\left(\omega_{0}\right)$ denoting the vacuum decay width of the QEMI with resonance frequency $\omega_{0}$ in free space; also, $\lambda^{k}\left(\omega ,D\right)$ denotes the directional Purcell factor of the QEMI placed at distance *D* from the TOPIN nanosphere surface, with k=z,x standing for along the *z* axis (tangential) and *x* axis (parallel) directions, respectively. We note that the Purcell factor along the *y* axis is as the Purcell factor along the *x* axis, since both the *x* and *y* directions correspond to tangential dipole moments of the QEMI with respect to the SNP. By applying the effective mode differential equation (EMDE) methodology [9], the probability amplitude dynamics are computed.

The effect of the altered continuum of electromagnetic modes due to the presence of the TOPIN nanosphere on the QEMI, which is located at distance *D* from the surface of the SNP of radius *R* is quantified after calculating the corresponding directional Purcell factors, $\lambda^{x}\left(\omega ,D\right)=\frac{\Gamma^{x}\left(\omega \right)}{\Gamma_{0}\left(\omega \right)}$ and $\lambda^{z}\left(\omega ,D\right)=\frac{\Gamma^{z}\left(\omega \right)}{\Gamma_{0}\left(\omega \right)}$, using a numerical electromagnetic Green’s tensor method [9,46]. The absorption cross-section of the SNP $\sigma_{abs}\left(\omega \right)$ given by [37]

(5) σabs(ω)=4πRωcImϵ(ω)+δR−1ϵ(ω)+δR+2

is determined by the electric field boundary conditions, with ϵ(ω) being the dielectric function of Bi${}_{2}$Se${}_{3}$. In Equation (5), $\delta_{R}$ denotes the term related to transitions between the delocalized topologically protected states perturbed by the incident light; it is given by [37]

(6) $$\delta_{R}=\frac{e^{2}}{6\pi \epsilon_{0}}\left(\frac{1}{2A-\omega R}+\frac{1}{2A+\omega R}\right)\;,$$

where A=0.3 eV nm, *R* stands for the TOPIN nanosphere radius, and the angular frequency of the incident light is denoted by ω. If $\delta_{R}=0$, then Equation (5) reduces to the case of a dielectric sphere in an uniform field. In addition, when $\delta_{R}=0$, within the quasi-static approximation, we compute the directional Purcell factors analytically, which are given by [47]

(7) λx(ω,D)=1+34cω3∑n=1Nn(n+1)(R+D)2n+4Iman

(8) λz(ω,D)=1+32cω3∑n=1N(n+1)2(R+D)2n+4Iman

with $a_{n}$ being the *n*-th multipole polarizability of the sphere defined by

(9) $$a_{n}=\frac{\epsilon (\omega )-1}{\epsilon (\omega )+(n+1)/n}R^{2n+1}\;.$$

In Equations (5) and (9), the dielectric function ϵ(ω) of Bi${}_{2}$Se${}_{3}$ is given by [37]

(10) $$\epsilon \left(\omega \right)=\sum_{i=a,\beta ,f}\frac{\omega_{pi}^{2}}{\omega_{0i}^{2}-\omega^{2}-i\gamma_{i}\omega }$$

including *a* and β transverse phonon contributions [48] and contributions from free charge carriers (labeled *f*) due to the bulk defects. The parameters for the three terms in Equation (10) are taken from a fit to experimental data on bulk. For Bi${}_{2}$Se${}_{3}$ [48], they read [37]: $\omega_{pa}\;=\;19.2$ THz, $\omega_{p\beta }=2.3$ THz, $\omega_{pf}=11.5$ THz, $\omega_{0a}=2$ THz, $\omega_{0\beta }=3.72$ THz, $\omega_{0f}=0$ THz, $\gamma_{a}=0.15$ THz, $\gamma_{\beta }=0.06$ THz, and $\gamma_{f}=0.24$ THz. We note that in Equations (7) and (8), N=10 is used. We also note that in this work, we deal with the near-field regime of the QEMI, for which its distance from the surface of the Bi${}_{2}$Se${}_{3}$ nanosphere is much smaller than the SPEM wavelength of the QEMI, D≪λ. Thus, we can use the electrostatic approximation.

## 3. Results and Discussion

We firstly discuss our findings when no delocalized topologically protected states, $\delta_{R}=0$, are considered in the absorption cross section $\sigma_{abs}\left(\omega \right)$ of Bi${}_{2}$Se${}_{3}$.

### 3.1. The Purcell Factors and SPEM Dynamics of a QEMI near a Bi${}_{2}$Se${}_{3}$ SNP with $\delta_{R}=0$

**Figure 2:**
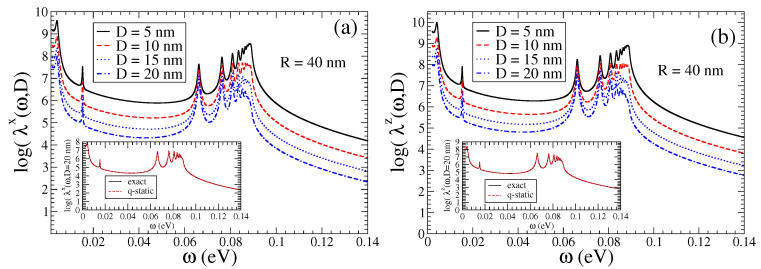
(color online) Purcell factor calculated numerically for a QEMI with transition dipole moment along (**a**) the x- and (**b**) z-axis next to a Bi${}_{2}$Se${}_{3}$ SNP, when $\delta_{R}=0$, as function of the QEMI distance *D* from the surface of a R=40 nm SNP; (insets) for a QEMI placed at D=20 nm from the surface of a Bi${}_{2}$Se${}_{3}$ SNP of radius R=40 nm compared to analytical results within the quasi-static approximation, using Equations (7) and (8).

In Figure 2, in the main panels, we present the Purcell enhancement factors $\lambda^{k}\left(\omega ,D\right)$, (k=z,x), for a QEMI placed at D=5,10,15,20 nm from a Bi${}_{2}$Se${}_{3}$ TOPIN nanosphere, with R=40 nm and $\delta_{R}=0$. An impressive increase in the decay rates over eight orders of magnitude is found, which apparently is not significantly affected by the distance *D* of the QEMI to the surface of the SNP. We note that distinct peaks arise above a frequency-independent—within the range shown—strong enhancement. We note that the Purcell factor increase dependence on the TOPIN nanosphere radius *R* for a QEMI placed at D=20 nm from the surface of the Bi${}_{2}$Se${}_{3}$ SNP is marginal (not shown here) for sphere radii in the range between 40 nm and 100 nm, as explicitly discussed in Ref. [36]. In the insets of the two panels of Figure 2, we compare the Purcell enhancement for a QEMI placed at D=20 nm from the surface of a Bi${}_{2}$Se${}_{3}$ SNP with R=40 nm obtained numerically with the electromagnetic Green’s tensor method [9,46] to the results obtained within the quasi-static approximation using Equations (7) and (8); obviously, the agreement between the two methods is very good, indicating in this case the validity of the quasi-static approximation.

**Figure 3:**
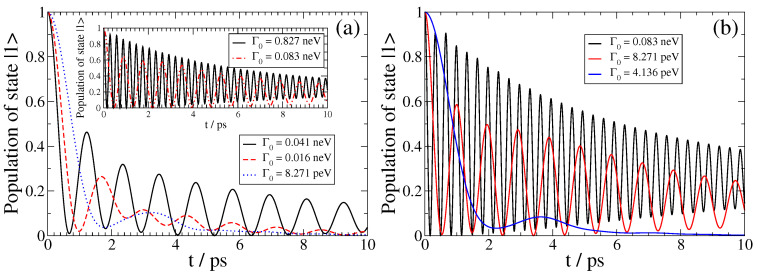
(color online) SPEM dynamics for a QEMI with $\omega_{0}=0.00414\;\mathrm{eV}$ (giving 1THz frequency) and transition dipole moment along the z-axis placed at (**a**) D=20 nm and (**b**) D=10 nm from a Bi${}_{2}$Se${}_{3}$ SNP with radius R=40 nm.

**Figure 4:**
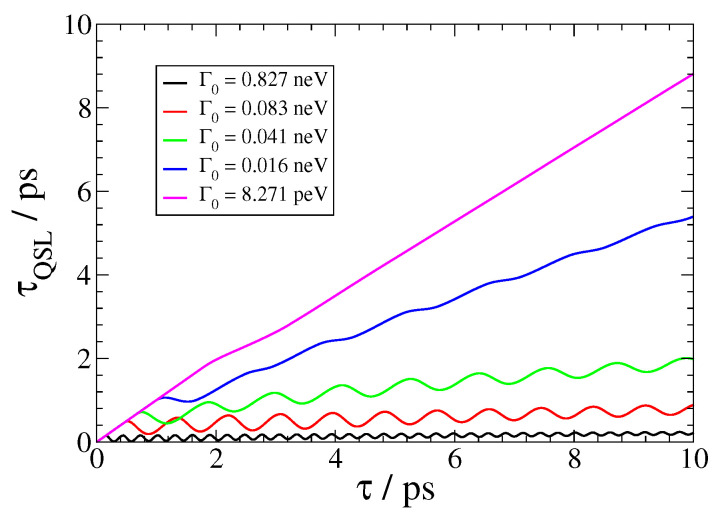
(color online) Quantum speed limit *τ_QSL_* for the SPEM dynamics of a QEMI, with *ω*_0_ = 0.00414 eV (1 THz frequency) with dipole moment along the z-axis, located at *D* = 20 nm from a Bi_2_Se_3_ SNP with radius *R* = 40 nm, as presented in Figure 2.

**Figure 5:**
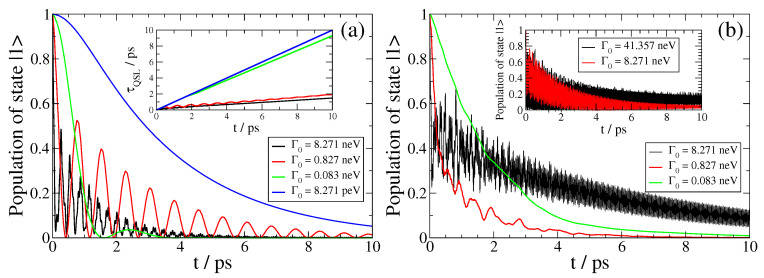
(color online) SPEM dynamics for a QEMI with transition dipole moment along the z-axis at D=20 nm from a Bi${}_{2}$Se${}_{3}$ SNP with radius R=40 nm for various $\Gamma_{0}$. (**a**): The dynamics for a QEMI with $\omega_{0}=0.06178\;\mathrm{eV}$ (16.002THz frequency) and the corresponding QSL of the dynamics (inset). (**b**): The dynamics for a QEMI with $\omega_{0}=0.086436\;\mathrm{eV}=20.907\;\mathrm{THz}$ for the main plot and $\omega_{0}=0.04\;\mathrm{eV}$ (9.672THz frequency) for the inset.

**Figure 6:**
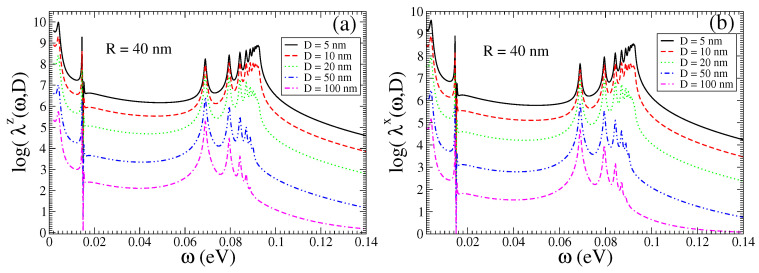
(color online) Numerically obtained Purcell enhancement factor for a QEMI with transition dipole moment along the (**a**) z- and (**b**) x-axis next to a Bi${}_{2}$Se${}_{3}$ SNP when $\delta_{R}\neq 0$ as function of the QEMI distance *D* from the surface of a R=40 nm SNP.

**Figure 7:**
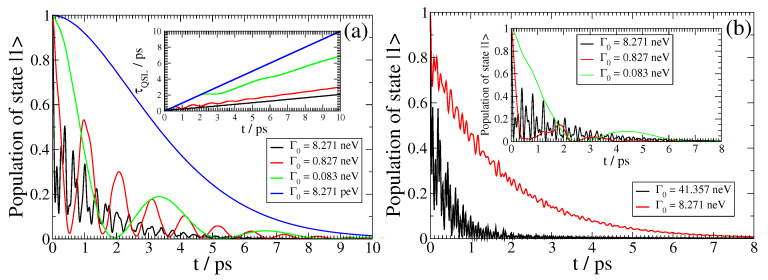
(color online) SPEM dynamics for a QEMI with transition dipole moment along the z-axis located at D=20 nm from a Bi${}_{2}$Se${}_{3}$ SNP with radius R=40 nm for various $\Gamma_{0}$ with $\delta_{R}\neq 0$. (**a**): The dynamics for a QEMI with $\omega_{0}=0.0689\;\mathrm{eV}$, corresponding to frequency 16.66THz, and the corresponding QSL of the dynamics (inset). (**b**): The dynamics for a QEMI with $\omega_{0}=0.04136\;\mathrm{eV}$, corresponding to frequency 10THz for the main plot and $\omega_{0}=0.084248\;\mathrm{eV}$, corresponding to 20.37THz for the inset.

We first focus on the SPEM dynamics of the QEMI near a Bi${}_{2}$Se${}_{3}$ sphere with R=40 nm, when $\delta_{R}=0$, presented in Figure 3, Figure 4 and Figure 5 below. We show results on the decay dynamics for state-of-the-art two-level QEMIs with transition frequencies $\omega_{0}$ in the terahertz regime, and vacuum decay time $\tau_{0}$ from nanoseconds to milliseconds, corresponding to a vacuum decay width $\Gamma_{0}=1/\tau_{0}$ in the peV to neV range of energy. As shown in Figure 2, there is no qualitative difference between the QEMI enhancement factors when the corresponding transition dipole moment is along the z-axis or along the x-axis; since the enhancement factors are largest for a QEMI with a transition dipole moment along the z-axis, we focus on such a case below, in order to maximize the effects under study. Furthermore, in Figure 6 and Figure 7, we investigate corresponding cases when $\delta_{R}\neq 0$.

In Figure 3, we show the SPEM dynamics of a QEMI with $\omega_{0}=0.00414$ eV placed at D=20 nm (left panel) and D=10 nm (right panel) from a Bi${}_{2}$Se${}_{3}$ TOPIN nanosphere with z-oriented transition dipole moment and R=40 nm for various $\Gamma_{0}$. In both cases, we observe that as the $\Gamma_{0}$ decreases, the non-Markovian features of the dynamics diminish gradually, in accordance with the fact that the light–matter interaction strength between the QEMI and the TOPIN nanosphere is proportional to the vacuum decay width. The dynamics point to an population transfer between the QEMI excited state and the continuum of electromagnetic modes as affected by the proximity to the TOPIN nanosphere in an oscillatory fashion, with slowly decreasing amplitude as the population gradually decays in the electromagnetic continuum. However, at both *D*, for the largest $\Gamma_{0}$, the QEMI population of the excited state decays only partially in the photonic continuum; we observe that in both cases, asymptotically in time, about 25% of the initial QEMI population remains trapped in the QEMI. This population trapping effect is a strong evidence that a hybrid bound state between the QEMI and the continuum of electromagnetic modes is created, which is a clear manifestation that the strong light-matter coupling regime has been achieved [10,24].

A semi-analytical estimate can be derived for the value of the trapped population when such a bound state is created. The QEMI population for t→∞, when a bound state with energy $E_{B}$ is formed, is given by

(11) $$P_{B}={\left[1+\int_{0}^{\infty }d\omega \frac{J(\omega )}{{\left(E_{B}-\omega \right)}^{2}}\right]}^{-2}.$$

We further refer to Refs. [24,49] for a detailed derivation of Equation (11); application of this equation to the cases with the largest $\Gamma_{0}$ in the panels of Figure 3 gives $P_{B}=0.249$ and $P_{B}=0.246$, respectively, which agrees very well with the numerically obtained values shown in this figure using the exact EMDE methodology.

We now study the non-Markovian behavior of the SPEM dynamics by using various measures of non-Markovianity [50,51,52,53] for the light–matter coupling between the QEMI and the continuum of electromagnetic modes as affected by the TOPIN nanosphere. We consider the following measures for the non-Markovianity of the quantum dynamics: The Breuer, Laine, Pillo (BLP) measure, N, defined in Ref. [50], which is based on the flow of information between the quantum system and the environment quantifying the non-Markovianity of the process by using the trace distance between two states, as well as the two Rivas, Huelga, Plenio (RHP) measures, $I^{E}$ and I, defined in Ref. [52], both quantifying the non-Markovian behavior of the entanglement dynamics by considering local trace-preserving completely positive maps. We note that the above three measures are equivalent when applied to a two-level QEMI coupled to photonic environments via different frequency-dependent couplings [54].

For the calculations, we use the time-dependent decay rate

(12) $$\gamma \left(t\right)=-2ℜ\left(\frac{{\dot{c}}_{1}\left(t\right)}{c_{1}\left(t\right)}\right)=-\frac{2}{|c_{1}\left(t\right)|}\frac{d}{dt}\left|c_{1}\left(t\right)\right|\;,$$

and the quantity

(13) $$F\left(t\right)=\frac{a^{2}e^{-\frac{3}{2}\Gamma \left(t\right)}+\frac{1}{2}{\left|b\right|}^{2}e^{-\frac{1}{2}\Gamma \left(t\right)}}{\sqrt{a^{2}e^{-\Gamma (t)}+{\left|b\right|}^{2}}}\;,$$

with

(14) $$\Gamma \left(t\right)=\int_{0}^{t}dt^{\prime }\gamma \left(t^{\prime }\right)\;.$$

Here, $a=\langle 1|\rho_{1}\left(0\right)|1\rangle -\langle 1|\rho_{2}\left(0\right)|1\rangle$ is the population difference, and $b=\langle 1|\rho_{1}\left(0\right)|0\rangle -\langle 1|\rho_{2}\left(0\right)|0\rangle$ is the coherence difference between the two arbitrary initial states. Then, the BLP measure [50] reads:

(15) $$N=-\max_{a,b}\int_{\gamma (t)<0}\gamma \left(t\right)F\left(t\right)dt\;.$$

Also, the RHP measures [52] can be obtained by

(16) $$I^{\left(E\right)}=-\int_{\gamma (t)<0}\gamma \left(t\right)e^{-\frac{1}{2}\Gamma \left(t\right)}dt\;,$$

and

(17) $$I=-\int_{\gamma (t)<0}\gamma \left(t\right)dt\;.$$

We show the N, $I^{E}$, and I non-Markovianity measure values in Table 1. The values increase as the oscillation period of the dynamics of the population decrease, since all three measure definitions are based only on the part of the dynamics, for which γ(t)<0. We note that the values between the three measures are not comparable, since each value is not normalized.

In Figure 4, we further show the QSL $\tau_{QSL}$ for the SPEM dynamics of a QEMI, with $\omega_{0}=0.00414$ eV and a transition dipole moment along the z-axis, placed at D=20 nm from the surface of a R=40 nm Bi${}_{2}$Se${}_{3}$ SNP. The minimal evolution of an open quantum system [55] is bounded by the QSL, and it is related to the non-Markovianity of the open quantum system dynamics by [56]

(18) $$\tau_{QSL}=\frac{t}{2\frac{\tilde{N}\left(t\right)}{1-|c_{1}{\left(t\right)|}^{2}}+1}\;,$$

with

(19) $$\tilde{N}\left(t\right)=0.5\int_{0}^{t}\left|\partial_{t^{\prime }}\left[c_{1}\left(t^{\prime }\right)c_{1}^{*}\left(t^{\prime }\right)\right]\right|dt^{\prime }+\left[c_{1}\left(t^{\prime }\right)c_{1}^{*}\left(t^{\prime }\right)\right]-1\;,$$

and *t* denoting the actual driving time of the open system. Thus, when $\tilde{N}=0$, Equation (18) implies that the QSL is equal to the actual driving time; otherwise, QSL always has a smaller value that the actual driving time, implying that by exploitation of the non-Markovianity of an open system, its actual dynamics can be accelerated in comparison to the corresponding dynamics of the system, which behave Markovian. This result is clear in Figure 4, since as the vacuum decay width $\Gamma_{0}$ becomes smaller, leading to Markovian dynamics, the corresponding $\tau_{QSL}\left(t\right)$ curve comes closer to the (conceivable) diagonal in the plot. On the other hand, as the dynamics become strongly non-Markovian, the corresponding $\tau_{QSL}\left(t\right)$ curve in Figure 4 comes closer to the horizontal axis; moreover, it becomes oscillatory, with increasing frequency and decreasing amplitude as the light–matter coupling strength between the QEMI and the TOPIN nanosphere becomes larger, since the vacuum decay width value increases. Independently from the coupling strength, the dynamics of the SPEM have an initial Markovian phase, as indicated by the $\tau_{QSL}\left(t\right)$ overlap for some initial time with the (conceivable) diagonal of the plot.

We now present the dynamics of SPEM of a QEMI with $\omega_{0}=0.06178\;\mathrm{eV}=16.002\;\mathrm{THz}$ and transition dipole moment along the z-axis placed at D=20 nm from the surface of Bi${}_{2}$Se${}_{3}$ SNP of radius R=40 nm. In the left panel of Figure 5, we show the dynamics for various vacuum decay widths, and in the inset of the same panel, we present the QSL of the corresponding dynamics. Interestingly, we find no trapping of population here, although the vacuum decay width takes values as large, or even larger, than in the case shown in the left panel of Figure 3. This is due to the smaller Purcell enhancement factor at this $\omega_{0}$, as shown in Figure 2, thus resulting effectively in weaker coupling between the QEMI with $\omega_{0}=16.002$ THz and the TOPIN nanosphere. We further observe that the dynamics of the QEMI population die out in an oscillatory manner, with decreasing oscillation frequency as the vacuum decay width decreases, clearly manifesting how the dynamics turn from non-Markovian to Markovian. These results are also manifested on the QSL of the corresponding dynamics, which is shown in the panel inset. We observe that the QSL of the dynamics approaches the (conceivable) diagonal of the graph as the vacuum decay width $\Gamma_{0}$ decreases; for the smallest $\Gamma_{0}$, it actually becomes identical to the diagonal, according to the corresponding Markovian population dynamics.

In the right panel of Figure 5, we present the SPEM dynamics of a QEMI with $\omega_{0}=0.086436\;\mathrm{eV}=20.907\;\mathrm{THz}$ and transition dipole moment along the z-axis placed at D=20 nm from the surface of an R=40 nm radius Bi${}_{2}$Se${}_{3}$ SNP. We present the QEMI population evolution for various $\Gamma_{0}$. We observe no population trapping under these coupling conditions, although the $\Gamma_{0}$ is as large, or even larger, than the case presented in the top panel of Figure 3, since the Purcell enhancement factor at this $\omega_{0}$ is smaller, as shown in the top panel of Figure 2, thus resulting effectively in weaker coupling. However, perhaps the most interesting feature of the SPEM dynamics of a QEMI with $\omega_{0}=20.907\;\mathrm{THz}$ for the $\Gamma_{0}$ studied is the fact that the QEMI population decays gradually into the electromagnetic continuum of modes modified by the presence of the TOPIN nanosphere, featuring only a minor oscillating flow of population between the QEMI and the continuum. In all cases of non-Markovian SPEM dynamics at similar $\Gamma_{0}$ presented above—except, of course, when population trapping occurs—the QEMI population wanes out into the continuum, while an oscillating flow of population (Rabi oscillations) between the QEMI and the continuum is taking place on top of the overall decay of the QEMI initial population into the continuum. Rabi oscillations on top of the QEMI population decay evolution are characteristic of a QEMI with transition frequency $\omega_{0}$ close to the central frequency of an isolated peak in the Purcell enhancement factor spectrum. Here, however, the QEMI transition frequency $\omega_{0}=20.907\;\mathrm{THz}$ is close to several peak central frequencies in the Purcell enhancement factor spectrum, as shown in Figure 2. This fact indicates that the SPEM dynamics of the QEMI are taking place under conditions of overlapping resonances in the TOPIN nanosphere, which leads to the characteristic for such a case of QEMI population evolution as observed here.

As noted above, the Purcell enhancement factor of a QEMI near a Bi${}_{2}$Se${}_{3}$ SNP, with $\delta_{R}=0$, is exceptionally high, being continuously within 1–25 THz frequency range, as shown in Figure 2. The question thus arising is whether the formation of a hybrid bound state between the QEMI and the electromagnetic continuum of modes, which is manifested by the QEMI (partial) population trapping effect, occurs only at QEMI transition frequencies that correspond to a phonon polariton resonance frequency of the TOPIN nanosphere, as manifested by the corresponding peak central frequency in the Purcell enhancement factor spectrum. This question is addressed in the inset of the right panel of Figure 5, where we present the SPEM dynamics for a QEMI with $\omega_{0}=0.04\;\mathrm{eV}$ (giving transition frequency 9.672THz). Although this QEMI transition frequency corresponds to no polariton resonance in the TOPIN nanosphere, the corresponding Purcell enhancement factor is very large, as shown in Figure 2, thus allowing for the formation of a hybrid bound state between the QEMI and the electromagnetic continuum of modes modified by the Be${}_{2}$Se${}_{3}$ SNP, as manifested by the QEMI population trapping shown in the inset of the right panel of Figure 5.

We note that the asymptotically in time values of the trapped population 0.13 and 0.04, for the dynamics with $\Gamma_{0}=41.357$ neV and $\Gamma_{0}=8.271$ neV, respectively, obtained using Equation (11), are in both cases in very good agreement with the numerical results shown in Figure 5. We also note that the coupling conditions for the QEMI with QEMI transition frequency 1 THz and $\Gamma_{0}=0.827$ neV (shown in Figure 3) are comparable to the coupling conditions for the QEMI with transition frequency 9.672 THz and $\Gamma_{0}=41.357$ neV (shown here), if one considers that here, the Purcell enhancement factor is about three orders of magnitude smaller than in the case when population trapping occurs as shown in Figure 3. We can thus conclude that in order to observe the formation of a hybrid bound state of a QEMI with the electromagnetic continuum of modes in presence of a TOPIN nanosphere, the proximity of the QEMI transition frequency to a polariton resonance of the Bi${}_{2}$Se${}_{3}$ SNP is secondary, as long as the actual Purcell enhancement factor at the QEMI transition frequency is large enough to imply strong coupling conditions between the QEMI and the electromagnetic mode continuum as modified by the TOPIN nanosphere.

Lastly, in Table 2, we present the values of the non-Markovianity measures for various vacuum decay widths and QEMI transition frequencies located at D=20 nm from a Bi${}_{2}$Se${}_{3}$ SNP of radius R=40 nm, when $\delta_{R}=0$. The values of the N, $I^{\left(E\right)}$, and I measures become smaller as the dynamics turn Markovian, reaching even zero as the SPEM of the QEMI decays practically in an exponential way.

### 3.2. The Purcell Factors and SPEM Dynamics of a QEMI near a Bi${}_{2}$Se${}_{3}$ SNP with $\delta_{R}\neq 0$

In this part, we discuss the results on the SPEM dynamics of a QEMI close to a Bi${}_{2}$Se${}_{3}$ SNP of radius R=40 nm, including in the absorption cross-section transitions between the delocalized topologically protected surface states perturbed by the incident light, i.e., $\delta_{R}\neq 0$.

In Figure 6, we show the Purcell enhancement factors $\lambda^{k}\left(\omega ,D\right)$, (k=z,x) computed with a Green’s tensor method [9], including the light-induced transitions between the delocalized topologically protected surface states of the TOPIN SNP, i.e., $\delta_{R}\neq 0$, for a QEMI placed at D=5,10,20,50,100 nm from a Bi${}_{2}$Se${}_{3}$ SNP with R=40 nm. An enhancement of the decay rates is observed at about eight orders of magnitude, comparable to the corresponding case with $\delta_{R}=0$ shown in Figure 2; it is not significantly affected by the distance of the QEMI to the surface of the TOPIN nanosphere, for *D* up to half the value of the SNP radius. However, the frequency-independent—within the range shown—strong enhancement is clearly reduced in comparison to the corresponding Purcell factors shown in the top two panels in Figure 2, indicating that the existence of the delocalized surface states induces a shielding effect regarding the polariton states in the inner of the TOPIN nanosphere. We note that in addition to the peak in the Purcell enhancement factor spectrum at about 0.0145 eV, in case of a Bi${}_{2}$Se${}_{3}$ SNP with R=40 nm for $\delta_{R}=0$, as shown in Figure 2, we now observe a peak and a dip, both strong and sharp, in the Purcell enhancement factor spectrum at about the same frequency, independently on the distance *D* of the QEMI from the TOPIN nanosphere, which are obviously related the light-induced transitions between the surface states in the SNP.

The Purcell enhancement factor dependence on the TOPIN nanosphere radius *R* for a QEMI located at D=20 nm from the surface of the Bi${}_{2}$Se${}_{3}$ SNP is not shown here, since it is discussed in detail in Ref. [36]. Here, we just note in brief that the influence of the TOPIN nanosphere radius is marginal on the enhancement factor for sphere radii in the range up to 100 nm, similarly to the corresponding cases when $\delta_{R}=0$. However, in the case of $\delta_{R}\neq 0$, the energy of the peak maximal values in the Purcell spectrum, corresponding to the polariton resonances of the Be${}_{2}$Se${}_{3}$ SNP, is slightly red-shifted as the sphere radius *R* increases, contrary to the $\delta_{R}=0$ case. In addition, the shift between the smaller *R* nanospheres is clearly distinguishable; this is not the case for the larger *R* SNPs. This observation indicates that as the surface over which the delocalized topologically protected surface states significantly increases, their physical effect is reduced, in accordance with the limit $\delta_{R}\to 0$ as R→∞ [37].

We focus on the SPEM dynamics of a QEMI with transition dipole moment along the z-axis placed at D=20 nm from the surface of Bi${}_{2}$Se${}_{3}$ SNP of radius R=40 nm. In the left panel of Figure 7, the QEMI dynamics with $\omega_{0}=0.0689\;\mathrm{eV}$ for various vacuum decay widths $\Gamma_{0}$ are shown; the corresponding QSL is shown in the inset of the same panel. Firstly, no population trapping is found here; secondly, the QEMI population decays in an oscillatory fashion, with decreasing oscillation frequency, as the vacuum decay width decreases, distinctly manifesting how the dynamics turn from non-Markovian to Markovian; both observations are qualitatively similar to the ones made from the results shown in Figure 5. These observations also hold for the QSL of the corresponding dynamical evolution, shown in the panel inset: the QSL curves comes closer to the (conceivable) diagonal of the plot as the vacuum decay width decreases.

In the inset of the right panel of Figure 7, we present the SPEM dynamics of a QEMI with $\omega_{0}=0.084248\;\mathrm{eV}$ and transition dipole moment along the z-axis located at D=20 nm from the surface of a R=40 nm radius Bi${}_{2}$Se${}_{3}$ SNP. We present the QEMI population dynamics for various vacuum decay widths. In the main right panel of Figure 7, the SPEM dynamics are shown for a QEMI with $\omega_{0}=0.04136\;\mathrm{eV}$, whose frequency is off-resonant to any polariton resonance in the TOPIN nanosphere, resulting in a small Purcell enhancement factor, as shown in Figure 6; thus, expectedly, we observe clear SPEM decaying dynamics in such a case, as manifested by the QEMI population evolution shown in the main right panel of Figure 7.

Further, in Table 3, we present the values of non-Markovianity measures for various vacuum decay widths $\Gamma_{0}$ and QEMI transition frequencies $\omega_{0}$ located at D=20 nm from a Bi${}_{2}$Se${}_{3}$ SNP of radius R=40 nm, when $\delta_{R}\neq 0$. The N, $I^{\left(E\right)}$, and I values increase as the dynamics turn non-Markovian.

**Figure 8:**
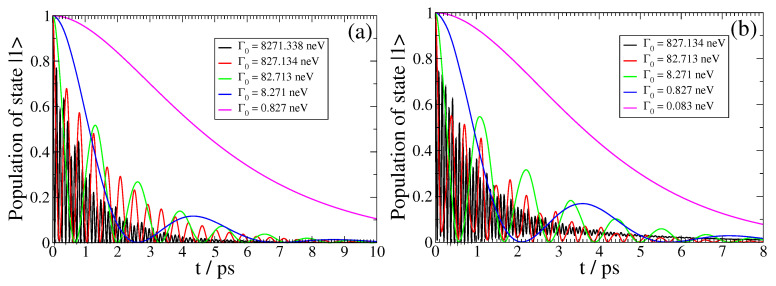
(color online) Spontaneous emission dynamics for a QE with $\omega_{0}=0.0689\;\mathrm{eV}=16.66\;\mathrm{THz}$ and z-oriented dipole moment at (**a**) D=100 nm and (**b**) D=50 nm from a Bi${}_{2}$Se${}_{3}$ SNP with radius R=40 nm for various $\Gamma_{0}$ with $\delta_{R}\neq 0$.

Lastly, in Figure 8, we investigate the influence of the decay width $\Gamma_{0}$ of the QEMI in relation to its distance *D* from the TOPIN nanosphere on the light–matter coupling strength. For this purpose, here, we present the SPEM dynamics for a QEMI with $\omega_{0}=0.0689$ eV and transition dipole moment along the z-axis placed at D=100 nm (left panel) and D=50 nm (right panel) from a Bi${}_{2}$Se${}_{3}$ SNP of radius R=40 nm. At this $\omega_{0}$ value, the corresponding Purcell factors differ by about an order of magnitude (not shown here). This fact raises the expectation that the QEMI decay dynamics at D=50 nm will show similar features to the QEMI dynamics at D=100 nm, when the corresponding QEMI decay widths differ by roughly a factor of 10. The results presented in the panels of Figure 8 confirm this expectation, clearly demonstrating that the operation within a particular light–matter interaction coupling regime can be achieved by varying the vacuum decay width of the QEMI, or alternatively, its distance from the TOPIN nanosphere, or, of course, both. This result holds also for the non-Markovian measure values and the QSL speedup as our calculations (not shown here) indicate.

## 4. Conclusions

In this work, we studied the light–matter coupling conditions of a QEMI near an SNP of Bi${}_{2}$Se${}_{3}$, a TOPIN material, with and without inclusion of the topologically protected delocalized surface states. By using computational electromagnetic methods, we computed in both cases the Purcell factor of a QEMI, with transition dipole moments with tangential and radial orientation with respect to the nanosphere, for various sphere radii and distance values between the QEMI and the surface of the SNP. We used experimental parameters for describing the optical properties of the Bi${}_{2}$Se${}_{3}$ nanosphere. We found exceptionally high enhancement factors, up to $10^{10}$, while the enhancement factors remained large, above $10^{5}$, within the whole frequency range 1–22 THz, for any transition dipole moment orientation, when no surface states are included in the calculations. We also showed the excellent agreement of the Purcell enhancement factors obtained by the quasi-static approximation with the values obtained using the computational electromagnetic method for different distances of the QEMI from the surface of the nanosphere, ranging up to several tens of nanometers. Moreover, when no surface states are included, we also found that the Purcell enhancement spectrum of Bi${}_{2}$Se${}_{3}$ features several narrow peaks, which become even narrower when $\delta_{R}\neq 0$.

We further investigated the dynamics of the SPEM of the QEMI in near a Bi${}_{2}$Se${}_{3}$ SNP for vacuum decay in the nanoseconds to milliseconds time range, when the QEMI is placed at half the SNP radius separation from the surface of the SNP and no delocalized surface states considered. For large vacuum decay width values, we observed that the dynamics of the SPEM are distinctively non-Markovian. Moreover, when the QEMI $\omega_{0}$ is resonant to an isolated polariton of the Bi${}_{2}$Se${}_{3}$ SNP, the dynamics of the SPEM manifest population transfer in a complete oscillatory manner (decaying Rabi oscillations) from the QEMI into the electromagnetic mode-continuum as altered by the presence of the TOPIN nanosphere and back. On the other hand, when the QEMI transition frequency is resonant to overlapping polaritons, the decay of the population of the QEMI into the continuum occurs with a population evolution featuring oscillations of minor amplitude superimposed to the overall decay. As the vacuum decay width decreases, the dynamics of the SPEM turn Markovian, with the population of the QEMI waning out in the continuum of electromagnetic modes practically in exponential way.

We also calculated the values of the BLP and RHP non-Markovianity measures for the cases studied; the measures values are decreasing as the underlying dynamics turn Markovian. Additionally, the QSL of the dynamics of the SPEM were computed; we found that when the light–matter interaction is strong, resulting in non-Markovian behavior, the dynamics can be significantly accelerated. Similar qualitative results were obtained for the dynamics of the SPEM of the QEMI in proximity to a Bi${}_{2}$Se${}_{3}$ nanosphere, when $\delta_{R}\neq 0$.

Lastly, for the shortest vacuum decay rates of a Bi${}_{2}$Se${}_{3}$ nanosphere, when $\delta_{R}=0$, we found that population remains partially trapped in the QEMI. This effect occurs in a QEMI independently of whether its transition frequency is close to the frequency of a polariton resonance in the TOPIN nanosphere. The effect of population trapping can be rationalized as the formation of a hybrid bound state between the QEMI and the electromagnetic continuum of modes modified by the Bi${}_{2}$Se${}_{3}$ SNP. A semi-analytical methodology for computing the amount of the initial population that remains trapped in such a hybrid bound state is in very good agreement with the asymptotically in time value of the population of the upper state obtained numerically. The formation of such a hybrid state is a manifestation of strong coupling conditions between the QEMI and the Bi${}_{2}$Se${}_{3}$ nanosphere, which also strongly correlates to large non-Markovianity measure values and possible significant quantum speedup of the dynamics. However, when $\delta_{R}\neq 0$, no population trapping is observed at conditions where such effects occurred when no surface states are included. Since the strength of the light–matter interaction depends on the relation between the distance of the QEMI from the TOPIN nanosphere and its vacuum decay width, as we find, the possibility of tuning the strength of the interaction on demand is clearly demonstrated.

In conclusion, evidently, a Bi${}_{2}$Se${}_{3}$ SNP can provide the necessary conditions for achieving strong light–matter coupling at the nanoscale, and this happens clearly in the terahertz regime of the spectrum; therefore, such a platform could serve as a single-photon source in this spectral region for quantum information processing. Furthermore, we have studied QEMIs with vacuum decay times which are realistic for a variety of nanoscale systems, thus rendering our results particularly interesting and valuable for developing novel quantum technologies.

## Figures and Tables

**Table 1:** Non-Markovianity measure values [50,52] for various $\Gamma_{0}$ for a QEMI with transition frequency 1 THz placed at D=10 and D=20 nm from a Bi${}_{2}$Se${}_{3}$ SNP of radius R=40 nm.

D=10 nm					
$\Gamma_{0}$/peV	82.713		8.271		4.136
N	16.56		5.14		0.11
$I^{E}$	34.05		10.31		0.22
I	92.59		39.04		1.00
D=20 nm					
$\Gamma_{0}$/peV	827.133	82.713	41.357	16.543	8.271
N	17.79	6.11	3.33	0.84	0.13
$I^{E}$	61.25	12.26	6.69	1.69	0.26
I	98.20	43.82	33.00	8.63	1.10

**Table 2:** Values of non-Markovianity measures [50,52] for various vacuum decay widths and QEMI transition frequencies located at D=20 nm from a Bi${}_{2}$Se${}_{3}$ SNP of radius R=40 nm.

Transition frequency 16.002 THz					
$\Gamma_{0}$ / neV	165.423	8.271	0.827	0.083	0.008
N		19.65	2.49	0.62	0
$I^{E}$		41.52	5.00	1.25	0
I		360.91	54.88	17.74	0
Transition frequency 20.907 THz					
$\Gamma_{0}$ / neV	165.423	8.271	0.827	0.083	
N		30.08	0.55	0	
$I^{E}$		60.42	1.10	0	
I		141.32	7.29	0	
Transition frequency 9.672 THz					
$\Gamma_{0}$ / neV	41.357			8.271	
N	101.47			36.45	
$I^{E}$	84.65			86.89	
I	672.90			352.99	

**Table 3:** Values of non-Markovianity measures [50,52] for various vacuum decay widths and QEMI transition frequencies $\omega_{0}$ located at D=20 nm from a Bi${}_{2}$Se${}_{3}$ SNP of radius R=40 nm, when $\delta_{R}\neq 0$.

$\omega_{0}=16.66$ THz					
$\Gamma_{0}$ / neV	8.271	0.827	0.083	0.008	
N	2.88	2.12	0.59	0	
$I^{E}$	5.77	4.23	1.18	0	
I	576.40	112.36	20.87	0	
$\omega_{0}=20.37$ THz					
$\Gamma_{0}$ / neV	8.271	0.827	0.083		
N	6.22	0.65	0.37		
$I^{E}$	12.45	1.30	0.74		
I	55.13	19.07	14.01		
$\omega_{0}=10$ THz					
$\Gamma_{0}$ / neV	41.357			8.271	
N	16.23			1.40	
$I^{E}$	32.46			2.81	
I	566.20			395.59	

## Data Availability

The data presented in this study are available upon reasonable request from the corresponding author.

## Abbreviations

The following abbreviations are used in this manuscript:

QEMI	Quantum Emitter
SPEM	Spontaneous Emission
TOPIN	Topological Insulator
SNP	Spherical Nanoparticle
BLP	Brueuer, Laine, Pillo
RHP	Rivas, Huelga, Plenio
QSL	Quantum Speed Limit

## References

[1] Baranov D.G., Wersäll M., Cuadra J., Antosiewicz T.J., Shegai T. (2018). Novel nanostructures and materials for strong light-matter interactions. ACS Photonics.

[2] Kolaric B., Maes B., Clays K., Durt T., Caudano Y. (2018). Strong light-matter coupling as a new tool for molecular and material engineering: Quantum approach. Adv. Quant. Technol..

[3] Fernández-Domínguez A.I., Bozhevolnyi S.I., Mortensen N.A. (2018). Plasmon-enhanced generation of nonclassical light. ACS Photonics.

[4] Zhang Q., Gong Q.-H., Gu Y. (2021). Enhanced Photon-Emitter Coupling in Micro/Nano Photonic Structures. IEEE J. Select. Top. Quant. Electron..

[5] González-Tudela A., Huidobro P.A., Martin-Moreno L., tejedor C., García-Vidal F.J. (2014). Reversible dynamics of single quantum emitters near metal-dielectric interfaces. Phys. Rev. B.

[6] Hakami J., Wang L., Zubairy M.S. (2014). Spectral properties of a strongly coupled quantum-dot–metal-nanoparticle system. Phys. Rev. A.

[7] Varguet H., Rousseaux B., Dzsotjan D., Jauslin H.R., Guérin S., Colas des Francs G. (2016). Dressed states of a quantum emitter strongly coupled to a metal nanoparticle. Opt. Lett..

[8] Li R.-Q., Hernángomez-Pérez D., García-Vidal F.J., Fernández-Domínguez A.I. (2016). Transformation optics approach to plasmon-exciton strong coupling in nanocavities. Phys. Rev. Lett..

[9] Thanopulos I., Yannopapas V., Paspalakis E. (2017). Non-Markovian dynamics in plasmon-induced spontaneous emission interference. Phys. Rev. B.

[10] Yang C.-J., An J.-H. (2017). Suppressed dissipation of a quantum emitter coupled to surface plasmon polaritons. Phys. Rev. B.

[11] Rousseaux B., Baranov D.G., Käll M., Shegai T., Johansson G. (2018). Quantum description and emergence of nonlinearities in strongly coupled single-emitter nanoantenna systems. Phys. Rev. B.

[12] Neuman T., Esteban R., Casanova D., García-Vidal F.J., Aizpurua J. (2018). Coupling of molecular emitters and plasmonic cavities beyond the point-dipole approximation. Nano Lett..

[13] Wang S., Scholes G.D., Hsu L.-Y. (2019). Quantum dynamics of a molecular emitter strongly coupled with surface plasmon polaritons: A macroscopic quantum electrodynamics approach. J. Chem. Phys..

[14] Yang C.-J., An J.-H., Lin H.-Q. (2019). Signatures of quantized coupling between quantum emitters and localized surface plasmons. Phys. Rev. Res..

[15] Wen S.-S., Huang Y.-G., Wang X.-Y., Liu J., Li Y., Quan X.-E., Yang H., Peng J.-Z., Deng K., Zhao H.-P. (2020). Bound state and non-Markovian dynamics of a quantum emitter around a surface plasmonic nanostructure. Opt. Express.

[16] Shahbazyan T.-V. (2022). Non-Markovian effects for hybrid plasmonic systems in the strong coupling regime. Phys. Rev. B.

[17] Liberal I., Engheta N. (2017). Zero-index structures as an alternative platform for quantum optics. Proc. Natl. Acad. Sci. USA.

[18] Thanopulos I., Karanikolas V., Iliopoulos N., Paspalakis E. (2019). Non-Markovian spontaneous emission dynamics of a quantum emitter near a MoS_2_ nanodisk. Phys. Rev. B.

[19] Thanopulos I., Karanikolas V., Paspalakis E. (2019). Non-Markovian spontaneous emission interference near a MoS_2_ nanodisk. Opt. Lett..

[20] Thanopulos I., Karanikolas V., Paspalakis E. (2021). Strong interaction of quantum emitters with a WS_2_ layer enhanced by a gold substrate. IEEE J. Select. Top. Quant. Electron..

[21] Ji F.-Z., Bai S.-Y., An J.-H. (2022). Strong coupling of quantum emitters and the exciton-polariton in MoS2 nanodisk. Phys. Rev. B.

[22] Deng Z.-S., Li L.-Y., You C.-L., Lu Y.-W., Liu J.-F. (2023). Effective Modes for a Strongly Coupled Quantum Emitter-MoS_2_ Nanodisk System. IEEE Photon. J..

[23] Koppens F.H.L., Chang D.E., García de Abajo F.J. (2011). Graphene Plasmonics: A Platform for Strong Light–Matter Interactions. Nano Lett..

[24] Thanopulos I., Karanikolas V., Paspalakis E. (2022). Spontaneous emission of a quantum emitter near a graphene nanodisk under strong light-matter coupling. Phys. Rev. A.

[25] Neuman T., Wang D.S., Narang P. (2020). Nanomagnonic Cavities for Strong Spin-Magnon Coupling and Magnon-Mediated Spin-Spin Interactions. Phys. Rev. Lett..

[26] Lu L., Joannopoulos J.D., Soljacic M. (2014). Topological Photonics. Nat. Photonics.

[27] Ozawa T., Price H.M., Amo A., Goldman N., Hafezi M., Lu L., Rechtsman M.C., Schuster D., Simon J., Zilberberg O. (2019). Topological photonics. Rev. Mod. Phys..

[28] Rider M.S., Palmer S.J., Rocock S.R., Xiao X.-F., Huidobro P.A., Giannini V. (2019). A perspective on topological nanophotonics: Current status and future challenges. J. Appl. Phys..

[29] Bello M., Platero G., Cirac J.I., González-Tudela A. (2019). Unconventional quantum optics in topological waveguide QED. Sci. Adv..

[30] García-Elcano I., González-Tudela A., Bravo-Abad J. (2020). Tunable and Robust Long-Range Coherent Interactions between Quantum Emitters Mediated by Weyl Bound States. Phys. Rev. Lett..

[31] Barik S., Karasahin A., Flower C., Cai T., Miyake H., DeGottardi W., Hafezi M., Waks E. (2018). A topological quantum optics interface. Science.

[32] Barik S., Karasahin A., Mittal S., Waks E., Hafezi M. (2020). Chiral quantum optics using a topological resonator. Phys. Rev. B.

[33] Qian Z.-Y., Li Z.-C., Hao H., Shan L.-X., Zhang Q., Dong J.-W., Gong Q.-H., Gu Y. (2021). Absorption reduction of large purcell enhancement enabled by topological state-led mode coupling. Phys. Rev. Lett..

[34] Hasan M.Z., Kane C.L. (2010). Topological Insulatots. Rev. Mod. Phys..

[35] Qi X.-L., Zhang S.C. (2011). Topological insulators and superconductors. Rev. Mod. Phys..

[36] Thanopulos I., Yannopapas V., Paspalakis E. (2022). Topological insulator nanoparticle for strong light-matter interaction in the terahertz. Opt. Lett..

[37] Siroki G., Lee D.K.K., Hayes P.D., Giannini V. (2016). Single-electron induced surface plasmons on a topological nanoparticle. Nat. Commun..

[38] Di Pietro P., Adhlakha N., Piccirilli F., Di Gaspare A., Moon J., Oh S., Di Mitri S., Spampinati S., Peruchi A., Lupi S. (2020). Terahertz Tuning of Dirac Plasmons in Bi_2_Se_3_ Topological Insulator. Phys. Rev. Lett..

[39] Rider M.S., Sokolikova M., Hanham S.M., Navarro-Cía M., Haynes P.D., Lee D.K.K., Daniele M., Cestelli Guildi M., Mattevi C., Lupi S. (2020). Experimental signature of a topological quantum dot. Nanoscale.

[40] Rider M.S., Buendía A.I., Abujetas D.R., Huidorbo P.A., Sánchez-Gil J.A., Giannini V. (2022). Advances and Prospects in Topological Nanoparticle Photonics. ACS Photonics.

[41] Chatzidakis G.D., Yannopapas V. (2020). Strong electromagnetic coupling in dimers of topological-insulator nanoparticles and quantum emitters. Phys. Rev. B.

[42] Castro-Enriquez L.A., Quezada L.F., Martin-Ruiz A. (2020). Optical response of a topological-insulator–quantum-dot hybrid interacting with a probe electric field. Phys. Rev. A.

[43] Karaoulanis D., Paspalakis E., Yannopapas V. (2021). Quantum interference near bismuth-chalcogenide microstructures. J. Opt. Soc. Am. B.

[44] Kyvelos N., Tsigaridas G., Paspalakis E., Yannopapas V. (2022). Quantum interference in spontaneous decay of a quantum emitter placed in a dimer of bismuth-chalcogenide microparticles. Photonics.

[45] Hamedi H.R., Ruseckas J., Yannopapas V., Karaoulanis D., Paspalakis E. (2023). Light-induced enhanced torque on double-V-type quantum emitters via quantum interference in spontaneous emission. Opt. Laser Technol..

[46] Yannopapas V., Vitanov N.V. (2007). Electromagnetic Green’s tensor and local density of states calculations for collections of spherical electromagnetic scatterers. Phys. Rev. B.

[47] Vielma J., Leung P.T. (2007). Nonlocal optical effects on the fluorescence and decay rates for admolecules at a mettalic nanoparticle. J. Chem. Phys..

[48] Butch N.P., Kirschenbaum K., Syers P., Sushkov A.B., Jenkins G.S., Drew H.D., Paglione J. (2010). Strong surface scattering in ultra-high mobility Bi_2_Se_3_ topological insulator crystalls. Phys. Rev. B.

[49] Shi T., Wu Y.-H., González-Tudela A., Cirac J.I. (2016). Bound States in Boson Impurity Models. Phys. Rev. X.

[50] Breuer H.-P., Laine E.-M., Piilo J. (2009). Measure for the degree of non-Markovian behaviour of quantum processes in open systems. Phys. Rev. Lett..

[51] Breuer H.-P., Laine E.-M., Piilo J., Vacchini B. (2016). Non-Markovian dynamics in open quantum systems. Rev. Mod. Phys..

[52] Rivas A., Huelga S.F., Plenio M.B. (2010). Entanglement and Non-Markovianity of quantum evolutions. Phys. Rev. Lett..

[53] Rivas A., Huelga S.F., Plenio M.B. (2014). Quantum non-Markovianity: Characterization, quantification and detection. Rep. Prog. Phys..

[54] Zeng H.-S., Tang N., Zheng Y.-P., Wang G.-Y. (2011). Equivalence of the measures of Non-Markovianity for open two-level systems. Phys. Rev. A.

[55] Deffner S., Lutz E. (2013). Quantum Speed Limit for Non-Markovian Dynamics. Phys. Rev. Lett..

[56] Xu Z.-Y., Luo S., Yang W.L., Liu C., Zhu S. (2014). Quantum speedup in a memory environment. Phys. Rev. A.

